# Investigating the influence of work-related stress on early labour market exit: the role of health

**DOI:** 10.1007/s10433-023-00778-7

**Published:** 2023-07-05

**Authors:** Lisa Toczek, Richard Peter

**Affiliations:** Department of Medical Sociology, Institute of the History, Philosophy and Ethics of Medicine, Faculty of Medicine, Ulm University, Parkstrasse 11, 89073 Ulm, Germany

**Keywords:** Effort–reward imbalance, Mediation analysis, Survival analysis, Longitudinal study, Self-rated health, Retirement

## Abstract

Early labour market exit of large birth cohorts will put pressure on the social security systems of many European countries, especially Germany. Despite political efforts, many people retire before the statutory retirement age. A well-known predictor of retirement is health, which, in turn, is influenced by psychosocial working conditions such as work-related stress. This study examined whether work stress is associated with early labour market exit. In addition, we investigated whether health mediates this association. Survey data of the German Cohort Study on Work, Age, Health and Work Participation (lidA study) were linked to register data from the Federal Employment Agency, from which information on labour market exit was obtained (*n* = 3636). During a 6-year follow-up period, Cox proportional hazard models were used to investigate the influence of work-related stress and health on early labour market exit, adjusting for sex, age, education, occupational status, income and supervisor behaviour. Work-related stress was measured by effort–reward imbalance (ERI). Additionally, a mediation analysis was conducted to investigate a possible mediation of the association between ERI and early labour market exit by self-rated health. Higher work-related stress increased the likelihood of early labour market exit (HR 1.86; 95% CI 1.19–2.92). However, when including health in the Cox regression, the significant effect of work-related stress disappeared. Poor health was a risk factor for early labour market exit (HR 1.49; 95% CI 1.26–1.76) independent of all confounders. The results of the mediation analysis showed that self-rated health mediated the association between ERI and early labour market exit. The balance between effort and reward at work plays a major role in improving the self-rated health of workers. Interventions that reduce work-related stress can help to improve health and thus to maintain older employees in the German labour market.

**Supplementary Information:**

The online version contains supplementary material available at 10.1007/s10433-023-00778-7.

## Introduction

The ageing population and the upcoming retirement of large birth cohorts in many industrialized countries will exacerbate the labour force. Several of those countries implemented special employment policies to encourage older workers to stay in employment longer or even require them to retire later by increasing the statutory pension age. With the societal need to retain employees in the labour market, early retirement will put pressure on the social security systems (European Commission 2020). Especially in Germany, the early retirement of the baby boomer generation might have a severe impact. In 2021, the average retirement age in Germany was 63.1 years for men and 63.2 years for women. Thus, the actual retirement age was below the statutory retirement of 65.7 years (OECD 2021). Leaving the labour market early can have numerous reasons. One main reason for early retirement is poor health (van Rijn et al. 2014, van den Berg et al. 2010b, Fisher et al. 2016). In turn, health is influenced by prevailing psychosocial working conditions such as work-related stress, which can also affect early retirement decisions (Toczek et al. 2022). The objective of this longitudinal study is to examine the association between work-related stress and early labour market exit among older employees in Germany and investigates whether health mediates this relationship.

An established theory of work-related stress is described by the model of effort–reward imbalance (ERI). It claims that the imbalance between the effort invested in work and the rewards received (money, esteem and career opportunities) can lead to emotional distress and strain reactions. The lack of reciprocity over time—high effort and low reward—can result in adverse health effects (Siegrist 1996). The relationship between the ERI model and health has been extensively studied (Siegrist et al. 2004). Work-related stress is associated with adverse health outcomes such as heart disease or depression (Kivimäki et al. 2012; Lunau et al. 2013; Rugulies et al. 2013).

Psychosocial working conditions such as work-related stress can be identified as push factors for retirement. In the previous studies, higher work-related stress (ERI) was found to predict early labour market exit (Hintsa et al. 2015, van den Berg et al. 2010a). A recent study in Sweden found a longitudinal association between lower ERI and working longer (Stengård et al. 2022). However, a previous review of Browne et al. (2019) concluded that there is insufficient evidence on the relationship between work-related stress and labour market exit. In addition, earlier studies did not find a significant association between work-related stress and early retirement (Robroek et al. 2013; Virtanen et al. 2014; Mäcken 2019). The previous research of how work-related stress is related to early labour market exit is inconclusive and scarce.

Health is also a push factor for retirement and a primary factor influencing exit from the labour market, although the evidence is mixed. The previous longitudinal studies have demonstrated that poor health is related to early labour market exit (van Rijn et al. 2014, van den Berg et al. 2010b, Fisher et al. 2016, Schuring et al. 2013, De Wind et al. 2017). However, the previous research also showed that good health can be a reason to leave the labour market earlier, especially for those who can financially afford it (Pond et al. 2010; Reeuwijk et al. 2017; De Wind et al. 2013). Thus, evidence for the influence of poor health on early retirement is inconsistent (Robroek et al. 2013, 2015; Reeuwijk et al. 2017).

Most studies investigated the direct effect of work stress on leaving the labour market with controlling for health outcomes. However, the previous research has shown that work stress can influence the health of employees, which, in turn, can influence their retirement decisions (Kivimäki et al. 2012; Lunau et al. 2013; Rugulies et al. 2013; Schuring et al. 2013; van Rijn et al. 2014; Fisher et al. 2016). Only few studies have considered mediating effects of health on the relationship between work-related stress and retirement (Mäcken 2019; Stengård et al. 2022). The previous study of Mäcken (2019) examined mediating effects of different health measures on the relationship between work stress (two different measures) and retirement age. However, the results of the mediating effects were mixed across different measures of work stress. No mediating effect of the association between ERI and retirement age by self-rated health was observed (Mäcken 2019). ERI has been investigated less extensively in the previous studies, or no relevant findings have been found. In the present study, we, therefore, focused on ERI as a measure for work stress, which contributes to the previous literature. Moreover, Stengård et al. (2022) and d'Errico et al. (2021) suspected that health mediates the influence of psychosocial work factors on retirement timing but did not investigate health as a mediator in their research. This study examines this assumption. Hence, this study investigates the mediating role of health in the relationship between work stress and early exit from the labour market.

Finally, the aim of the present study is to close the research gap on work stress, health and early labour market exit in the German context. We investigated the relationship between work-related stress (ERI) and early labour market exit. In addition, we tested for a possible mediation of the association between ERI and early labour market exit by self-rated health.

## Methods

### Study sample

In the present study, data from the German Cohort Study on Work, Age, Health and Work Participation (lidA study) were used (Hasselhorn et al. 2014). LidA collected data via computer-assisted personal interviews (CAPI) of individuals of the baby boomer generation (born either in 1959 or 1965). In 2011, the baseline survey comprised 6585 interviews (Schröder et al. 2013). LidA included employed individuals who were required to pay social security contribution, which, in turn, excluded self-employed, freelancers or civil servants (Hasselhorn et al. 2014; Schröder et al. 2013). In addition to survey data, annual employment information could be collected through linkage with register data from the Federal Employment Agency. Respondents of lidA were asked to give their written consent to the inclusion of this register data, which was retrieved from the annual reports submitted by employers to the social security authority. By linking survey data with register data, valid information about labour market participation of each individual could be obtained (Schröder et al. 2013).

At baseline in 2011, 6585 interviews were conducted. A total of 4921 individuals gave written consent to the linkage of the survey data with the register data, and of these, 4750 individuals were in employment subject to social security contributions. IEB data could not be provided for 25 individuals. Respondents with missing values for the main variables in the survey data were excluded (*n* = 1089). The socio-demographic and socio-economic characteristics of the excluded individuals are displayed in Additional file 1: Table S1. The selectivity analysis showed that significantly more women and individuals with higher occupational status and higher income were included in the sample for analysis compared to the excluded individuals. Therefore, 3636 individuals who were employed at baseline (2011) and who could be linked to register data were included in the present study.

### Measures

#### Early labour market exit

We measured early labour market exit using information about labour market participation from the register data. The event of early labour market exit was determined when no information could be found in the employment register data of the person concerned. Based on the register data, the time of labour market exit can be determined to the day. A follow-up period was defined as time, in days, from the interview of the baseline survey until an event (i.e. early labour market exit) or censoring occurred. The follow-up period covers the time from 15 June 2011 until 31 December 2017. During the 6-year follow-up period, the time in days until labour market exit was determined, if applicable. If no event in the register data was observed, individuals were censored.

#### Work-related stress

Work-related stress was measured at baseline through the model of effort–reward imbalance (Siegrist 1996). The model assumes that a permanent imbalance of efforts and rewards increases the risk of adverse health outcomes. The ERI questionnaire measures the subscale “effort” with six items and the subscale “reward” with 11 questions. A ratio is computed by dividing both Likert scale subscales. In addition, a weighting factor of 6/11 is added to the denominator (subscale reward) to adjust for the varying numbers of items in the subscales. The ratio value of 1 can be defined as balanced efforts and rewards. Values close to zero indicate low effort and high reward, in contrast with values above 1.0 which indicate high ERI imbalance (Siegrist 1996; Siegrist et al. 2004). Therefore, higher ERI values indicate an increase in work-related stress.

#### Self-rated health

Health was assessed at baseline by one question of the Short-Form Survey (SF-12) (Nübling et al. 2006). The respondence were asked: “How would you rate your general health status?” with answers on a 5-point Likert scale ranging from (1) very good to (5) bad. Therefore, higher values indicate poorer health.

### Potential confounders

#### Sex

Sex was assumed to be a confounder. It was defined as (1) male and (2) female.

#### Year of birth

Age was assessed through the year of birth of the study cohorts, born in (1) 1959 or (2) 1965. During the 6-year follow-up period, the age of the older cohort ranged from 52 to 58 years and of the younger cohort from 46 to 52 years old.

#### Overcommitment

As a component of the ERI model, overcommitment was included in the analysis. It relates to individual differences regarding how people experience the imbalance between effort and reward (Siegrist et al. 2004).

#### Socio-economic indicators

Indicators of socio-economic status were included. Education considered information regarding both school education and vocational training (Jöckel et al. 1998). The combined categories included: (1) low, (2) intermediate and (3) high education. Occupational status was measured in four categories: (1) unskilled worker, (2) skilled worker, (3) middle management worker and (4) professional. Information regarding income was determined as current net monthly income with categories from: (1) low, (2) middle–low, (3) middle–high and (4) high income.

#### Supervisor behaviour

An item from the German version of COPSOQ (Nübling et al. 2005) was applied to measure supervisor behaviour. The respondents were asked to assess the extent to which their supervisor: “provides good development opportunities for the staff,” “gives high priority to job satisfaction,” “plans the work well” and “resolves conflicts well.” The response was given in a 5-point Likert scale: 1 = to a very large extent, 2 = to a large extent, 3 = to some extent, 4 = to a small extent and 5 = to a very small extent. To ease interpretation, the supervisor scale was reversed. Therefore, higher values on the scale indicate higher appraisal of the supervisor behaviour.

All information about covariates and potential confounders were taken from the baseline survey in 2011.

### Statistical analysis

First, descriptive statistics were used to describe the sample distribution for all baseline characteristics in 2011 (Table 1).

**Table 1:** Distribution of characteristics from the baseline survey 2011 (n = 3636)

Variables	Categories	Statistics N (%) or means (SD^a^)
Sex	Male	1774 (48.8)
	Female	1862 (51.2)
Year of birth	1959	1563 (43.0)
	1965	2073 (57.0)
Education	Low	853 (23.5)
	Intermediate	2012 (55.3)
	High	771 (21.2)
Occupational status	Unskilled workers	680 (18.7)
	Skilled workers	1365 (37.5)
	Middle management workers	1456 (40.0)
	Professionals	135 (3.7)
Income	Low	755 (20.8)
	Middle–low	1605 (44.1)
	Middle–high	828 (22.8)
	High	448 (12.3)
Self-rated health	Very good	445 (12.2)
	Good	1547 (42.5)
	Satisfactory	1159 (31.9)
	Not so good	406 (11.2)
	Bad	79 (2.2)
Work-related stress (ERI)		0.578 (0.27)
Overcommitment		13.44 (4.28)
Supervisor behaviour	53.80 (22.83)	

^a^ SD = standard deviation

Second, a survival analysis was used to estimate the “survival time” until leaving the labour market (early labour market exit). Individuals were censored if they did not leave the labour market at the end of the follow-up (31 December 2017). Cox proportional hazard models were used to investigate predictors of early labour market exit (Cox and Oakes 1984). We tested three models stepwise adjusting for possible confounders. Model 1 included work-related stress (ERI) as only predictor on early labour market exit, model 2 additionally included self-rated health and model 3, a full model, additionally controlled for possible confounders. We reported hazard ratios (HR) and 95% confidence intervals (CI). In the Cox proportional hazard model, an HR value > 1 indicates an increased likelihood of early labour market exit.

**Fig. 1:**
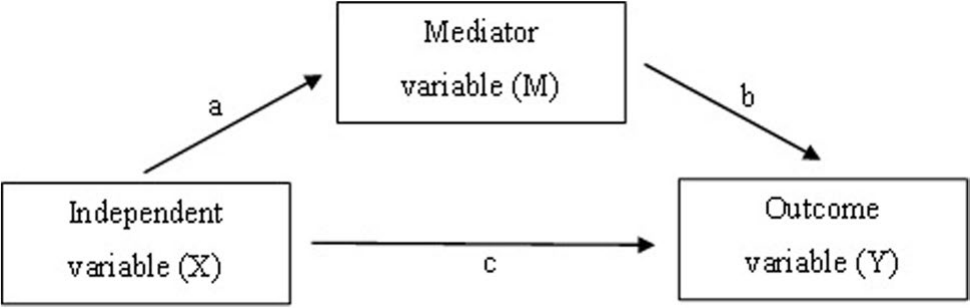
Conceptual diagram of a simple mediation model

Third, for the mediation analysis, early labour market exit was measured during the 6-year follow-up period until 2017 and categorized into: (0) no early labour market exit and (1) early labour market exit. A simple mediation model is displayed in Fig. 1. The independent variable X (e.g. work-related stress) can influence the outcome variable Y (e.g. early labour market exit) by two pathways. A direct pathway is demonstrated by path c and an indirect pathway over the mediator variable M (e.g. self-rated health) by path a and path b. The outcome variable in our mediation model is binary, and Hayes’ process macro can be used to test for the potential mediating role of self-rated health in the association between ERI and early labour market exit (Hayes 2022). Logistic regression analysis was conducted, estimating the effects on Y. OLS regression analysis was used for the direct effect of X on M. The direct and indirect effects of X on Y are on log-odds metric, as the effect of self-rated health (M) on the early labour market (Y) is a logistic regression coefficient. Furthermore, the mediation analysis was controlled for possible confounders. The log-odds can be transformed through exponentiation into odds ratios (ORs) (Hayes 2022). The effect estimates were accompanied by 95% percentile bootstrap confidence intervals (BootCIs) based on 5000 repeating samples.

All statistical analyses were performed using the statistic software IBM SPSS 28. A *p* value of < 0.05 was considered statistically significant.

## Results

### Descriptive results

In Table 1, the sample characteristics at baseline are presented (*n* = 3636). Male respondents accounted for 48.8% (*n* = 1774) while 51.2% were female (*n* = 1862). The mean and standard deviation of work-related stress (ERI) was 0.578 (± 0.27). The majority of respondents were in good (42.5%) or satisfactory (31.9%) health. The percentage of respondents in very good health were 12.2% (*n* = 445) and in not so good health 11.2% (*n* = 406). Only 2.2% of the respondents were in bad health (*n* = 79).

### Survival analysis

Until follow-up, 172 individuals were identified having an event, while 3464 were censored. Therefore, during the 6-year follow-up period, 172 individuals were observed to leave the labour market early. Table 2 displays the results of the Cox proportional hazard model, estimating the influence of work-related stress and self-rated health on the likelihood of early labour market exit. The hazard ratios of early labour market exit with 95% confidence intervals are derived from the Cox proportional hazard model. A significant effect of work-related stress on early labour market exit was found (model 1). High work-related stress increased the risk of early labour market exit (HR 1.86; 95% CI 1.19–2.92). However, including self-rated health as predictor in the Cox regression (model 2), no significant association could be observed of ERI on early labour market exit. Self-rated health was associated with early labour market exit, with an approximately 60% higher risk of early labour market exit for an increase in one unit in self-rated health. After adjustment for socio-demographic indicators, socio-economic indicators and supervisor behaviour (model 3), again, poor self-rated health was significantly associated with early labour market exit (HR 1.5; 95% CI 1.26–1.76). No statistically significant association between work-related stress and early labour market exit was observed in the full model.

**Table 2:** Influence of work-related stress (ERI) and self-rated health^a^ assessed at baseline, on the likelihood of early labour market exit during the 6-year follow-up among older German employees (n = 3636). Significant HR^b^ with 95% CI^c^ are marked in bold (*p* < 0.05)

	Variables	HR	95% CI
Model 1^d^	Work-related stress	**1.86**	**1.19–2.92**
Model 2^e^	Work-related stress	1.21	0.73–2.02
	Self-rated health	**1.61**	**1.37–1.88**
Model 3^f^	Work-related stress	0.86	0.46–1.60
	Self-rated health	**1.49**	**1.26–1.76**
	Sex	1.03	0.72–1.49
	Year of birth	**0.91**	**0.87–0.96**
	Overcommitment	1.03	0.99–1.07
	Education		
	Low (ref.)		
	Intermediate	1.12	0.78–1.63
	High	0.71	0.41–1.23
	Occupational status		
	Unskilled workers (ref.)		
	Skilled workers	0.67	0.44–1.02
	Middle management workers	0.66	0.42–1.05
	Professionals	1.30	0.61–2.79
	Income		
	Low (ref.)		
	Middle–low	0.80	0.52–1.21
	Middle–high	1.01	0.59–2.31
	High	1.15	0.57–2.31
	Supervisor behaviour	**0.99**	**0.98–0.99**

^a^Higher values indicate poorer health

^b^HR = hazard ratio

^c^95% CI = 95% confidence interval

^d^Model 1 included work-related stress (ERI)

^e^Model 2 additionally included self-rated health

^f^Model 3 additionally controlled for possible confounders

### Mediation analysis

The results of the mediation analysis are presented in Table 3. The direct effect from work-related stress to self-rated health was positive and significant, indicating that individuals with higher ERI are more likely to have poorer self-rated health. The direct effect from work-related stress to early labour market was positive but not significant. The direct effect from health to early labour market exit was positive and significant (*b* = 0.4829; SE = 0.0842; *p* < 0.05), indicating that individuals with poorer health were more likely to exit the labour market early. The indirect effect of work-related stress on early labour market exit through self-rated health was significant. Therefore, the criteria for mediation were fulfilled. For one unit increase in work-related stress, the odds of an early labour market exit increased by a factor of 1.60 via a decrease in self-rated health.

**Table 3:** Regression coefficients of mediation analysis^a^ with work-related stress (X) as predictor of the outcome variable early labour market exit (Y) and the mediator variable self-rated health^b^ (M)

	Coefficients (b)	SE^c^ or BootSE^d^	95% CI^e^ or 95% BootCI^f^
Direct effect on M			
X → M	0.9605	0.0536	0.8553; 1.0656
Direct effect on Y^g^			
M → Y	0.4427	0.0863	0.2736; 0.6117
X → Y	0.2562	0.2690	−0.2711; 0.7835
Indirect effect^g^			
X → Y	0.4252	0.0973	0.2412; 0.6222

^a^Analysis controlled for sex, year of birth, education, occupational status and income

^b^Higher values indicate poorer health

^c^SE = standard error

^d^BootSE = bootstrap standard error

^e^95% CI = confidence interval

^f^95% BootCI = bootstrap confidence intervals

^g^Coefficients were expressed in a log-odds metric

## Discussion

The aim of this study was to investigate the relationship between work-related stress and early labour market exit in Germany and additionally to examine possible mediating effects of self-rated health in this association. Our findings showed that the likelihood of early labour market exit increased with higher ERI and poor health. The relationship between work-related stress and early labour market exit was mediated by self-rated health. This indicates that not only a balance between effort and reward at work but also a good health status of a person are important factors to maintain older employees in the German labour market.

The previous research on the relationship between work-related stress and early labour market exit is scarce, and evidence is inconclusive. In contrast with earlier research, the present study found an association between work-related stress and retirement decisions (Mäcken 2019; Robroek et al. 2013; Virtanen et al. 2014). Higher ERI was found to increase the likelihood of early retirement, which was in line with some previous studies (Hintsa et al. 2015, Stengård et al. 2022, van den Berg et al. 2010a). In addition, the measurements of the outcome variable retirement varied considerably. In most studies, other pathways of retirement such as unemployment or disability pensions were included. Retirement, which can be identified as a more voluntary pathway compared to unemployment or disability pension, was either not measured or no significant results were found (Browne et al. 2019; Knardahl et al. 2017; Juvani et al. 2014; Robroek et al. 2013; Mäcken 2019). The different findings of the previous research may, therefore, be explained by different measurements of labour market exit.

In the present study, individuals with no information in the register data were defined as leaving the labour market early. Given the long follow-up period, early exit from the labour market is most likely permanent and not just temporary. Therefore, early labour market exit in this age group can most likely considered to be early retirement (Tisch 2015).

The findings of this study with regard to the relationship between health and early labour market exit are in line with earlier research (van Rijn et al. 2014, van den Berg et al. 2010b, Fisher et al. 2016, Schuring et al. 2013, De Wind et al. 2017). Poor self-rated health increased the likelihood of early labour market exit. A previous meta-analysis of van Rijn et al. (2014) also found poor self-rated health to be a risk factor of exit from paid employment. However, the authors found that this association varied across the pathways of labour market exit, with early retirement being less pronounced compared to disability pension or unemployment (van Rijn et al. 2014). Moreover, other studies found no association between poor health and early retirement, but regarding disability pension (Robroek et al. 2013, 2015). Similarly to prior research on ERI and early labour market exit, the varied outcomes observed in the previous studies examining the link between health and retirement may be attributed to the diverse retirement metrics employed.

The findings of the Cox proportional hazard model showed that work-related stress is associated with early labour market exit. However, adding self-rated health to the Cox regression, the association between work-related stress and early labour market exit disappeared, suggesting a mediating role of self-rated health. Mediation analysis showed no direct effect of work-related stress on early labour market exit when controlled for self-rated health. Nonetheless, an indirect effect of work-related stress via self-rated health on early labour market exit could be observed. Therefore, in the present study, self-rated health significantly mediated the relationship between ERI and early labour market exit, in contrast with the previous research that either found no mediating effect of health in the relationship between ERI and retirement (Mäcken 2019) or did not investigate health as a mediator in the relationship between psychosocial work factors and retirement timing (Stengård et al. 2022; d'Errico et al. 2021).

The present study has some limitations. The generalizability of our results is limited to German employees subject to social security contribution and therefore excluding civil servants, self-employed and freelancers. However, this study is highly representative of German employees subject to social security contributions of the two birth cohorts (1959 and 1965). Thus, one should be careful in generalizing our findings based on the German labour market to countries with different welfare state regimes. Nevertheless, social security systems of many other European countries will face more pressure as the workforce ages and more early labour market exit approaches. Therefore, our findings could be both useful and worthwhile for these countries with issues similar to those in Germany. Furthermore, the selectivity analysis showed minor differences between the study sample and the excluded individuals. Therefore, bias cannot be completely excluded. In addition, work-related stress was only measured at baseline. If employees have been exposed to more work-related stress after the baseline survey, this can lead to underestimation. However, a previous study showed that ERI at baseline predicted of ERI 3 years later quite well. Thus, underestimation seems to be unlikely (Peter et al. 2016). In addition, we did not consider other pathways to retirement because the register data contained limited information that would not have allowed for a longitudinal approach. In the present study, however, we focused on survival analysis and, therefore, relied on longitudinal information on early labour market exit from the register data. Despite these limitations, this study has several strengths. The linkage of survey data with register data is a major strength. Register data provide daily information about labour market participation of each individual. Moreover, the longitudinal character of our data is a strength. We utilized a 6-year follow-up period during which we collected daily updates on employment status for all the persons listed in the register data. Longitudinal data allowed us to analyse the impact of work-related stress on early labour market exit, with additionally examining the role of health. Another strength of this study is that the register data contain reliable and valid information, which was gathered from employers’ yearly reports submitted to the social security authorities. Hence, the register data are not affected by possible recall bias compared to self-reported data. Further research could consider additional work-related factors and different pathways of labour market exit, while not overlooking health as a potential mediator.

## Conclusion

This study contributes to the previous research by showing that older German employees tend to retire early when work creates stressful conditions, particularly due to the imbalance between effort and reward. Furthermore, higher work-related stress contributes substantially to poorer health among older employees. Therefore, interventions to reduce work-related stress can help to improve health and thus reduce early labour market exit.

## Supplementary Information

Below is the link to the electronic supplementary material.

**Additional file 1**: **Table S1** Distribution of socio-demographic and socio-economic indicators of the excluded individuals in % (*n* = 1089).

## Data Availability

The research data contain social security information. Due to legal regulations in Germany, it is not permitted to share data with social security information. Hence, the research data are confidential.

## References

[CR1] Browne P, Carr E, Fleischmann M, Xue B, Stansfeld SA (2019). The relationship between workplace psychosocial environment and retirement intentions and actual retirement: a systematic review. Eur J Ageing.

[CR2] Cox DR, Oakes D (1984) Analysis of survival data. Monographs on Statistics and Applied Probability, v.21. CRC Press, Boca Raton

[CR3] De Wind A, Burr H, Pohrt A, Hasselhorn HM, van der Beek AJ, Rugulies R (2017). The association of health and voluntary early retirement pension and the modifying effect of quality of supervision: Results from a Danish register-based follow-up study. Scand J Public Health.

[CR4] De Wind A, Geuskens GA, Reeuwijk KG, Westerman MJ, Ybema JF, Burdorf A, Bongers PM, van der Beek AJ (2013). Pathways through which health influences early retirement: a qualitative study. BMC Public Health.

[CR5] d'Errico A, Burr H, Pattloch D, Kersten N, Rose U (2021). Working conditions as risk factors for early exit from work-in a cohort of 2351 employees in Germany. Int Arch Occup Environ Health.

[CR6] European Commission (2020) The 2021 ageing report: underlying assumptions and projection methodologies. Publications Office

[CR7] Fisher GG, Chaffee DS, Sonnega A (2016). Retirement timing: a review and recommendations for future research. WORKAR.

[CR8] Hasselhorn HM, Peter R, Rauch A, Schröder H, Swart E, Bender S, Du Prel J-B, Ebener M, March S, Trappmann M, Steinwede J, Müller BH (2014). Cohort profile: the lidA Cohort Study—a German cohort study on work, age, health and work participation. Int J Epidemiol.

[CR9] Hayes AF (2022) Introduction to mediation, moderation, and conditional process analysis. A regression-based approach. Methodology in the social sciences, 3rd edn. The Guilford Press, New York

[CR10] Hintsa T, Kouvonen A, McCann M, Jokela M, Elovainio M, Demakakos P (2015). Higher effort-reward imbalance and lower job control predict exit from the labour market at the age of 61 years or younger: evidence from the English Longitudinal Study of Ageing. J Epidemiol Commun Health.

[CR11] Jöckel KH, Babitsch B, Bellach BM, Bloomfield K, Hoffmeyer-Zlotnik J, Winkler J, Wolf C (1998) Messung und Quantifizierung soziodemographischer Merkmale in epidemiologischen Studien. [Measurement and quantification of sociodemographic characteristics in epidemiological studies.]. https://www.rki.de/DE/Content/Gesundheitsmonitoring/Studien/Methodik/Empfehlungen/empfehlungen_node.html. Accessed 08 Jul 2021

[CR12] Juvani A, Oksanen T, Salo P, Virtanen M, Kivimäki M, Pentti J, Vahtera J (2014). Effort-reward imbalance as a risk factor for disability pension: the Finnish Public Sector Study. Scand J Work Environ Health.

[CR13] Kivimäki M, Nyberg ST, Batty GD, Fransson EI, Heikkilä K, Alfredsson L, Bjorner JB, Borritz M, Burr H, Casini A, Clays E, de Bacquer D, Dragano N, Ferrie JE, Geuskens GA, Goldberg M, Hamer M, Hooftman WE, Houtman IL, Joensuu M, Jokela M, Kittel F, Knutsson A, Koskenvuo M, Koskinen A, Kouvonen A, Kumari M, Madsen IEH, Marmot MG, Nielsen ML, Nordin M, Oksanen T, Pentti J, Rugulies R, Salo P, Siegrist J, Singh-Manoux A, Suominen SB, Väänänen A, Vahtera J, Virtanen M, Westerholm PJM, Westerlund H, Zins M, Steptoe A, Theorell T (2012). Job strain as a risk factor for coronary heart disease: a collaborative meta-analysis of individual participant data. Lancet.

[CR14] Knardahl S, Johannessen HA, Sterud T, Härmä M, Rugulies R, Seitsamo J, Borg V (2017). The contribution from psychological, social, and organizational work factors to risk of disability retirement: a systematic review with meta-analyses. BMC Public Health.

[CR15] Lunau T, Wahrendorf M, Dragano N, Siegrist J (2013). Work stress and depressive symptoms in older employees: impact of national labour and social policies. BMC Public Health.

[CR16] Mäcken J (2019). Work stress among older employees in Germany: Effects on health and retirement age. PloS one.

[CR17] Nübling M, Andersen HH, Mühlbacher A (2006) Entwicklung eines Verfahrens zur Berechnung der körperlichen und psychischen Summenskalen auf Basis der SOEP-Version des SF 12 (Algorithmus) [Development of a procedure for calculating the physical and mental sum scales based on the SOEP version of the SF 12 (algorithm)]. https://www.diw.de/documents/publikationen/73/diw_01.c.44987.de/diw_datadoc_2006-016.pdf. Accessed 18 May 2021

[CR18] Nübling M, Stößel U, Hasselhorn H-M, Michaelis M, Hofmann F (2005) Methoden zur Erfassung psychischer Belastungen - Erprobung eines Messinstrumentes (COPSOQ). Schriftenreihe der Bundesanstalt für Arbeitsschutz und Arbeitsmedizin Forschung, vol 1058. Wirtschaftsverl. NW Verl. für Neue Wiss, Bremerhaven

[CR19] OECD (2021) Ageing and Employment Policies - Statistics on average effective age of labour market exit. Pensions at a Glance. https://stats.oecd.org/Index.aspx?QueryId=111939

[CR20] Peter R, March S, Du Prel J-B (1982). Are status inconsistency, work stress and work-family conflict associated with depressive symptoms? Testing prospective evidence in the lidA study. Soc Sci Med.

[CR21] Pond R, Stephens C, Alpass F (2010). How health affects retirement decisions: three pathways taken by middle-older aged New Zealanders. Ageing Soc.

[CR22] Reeuwijk KG, van Klaveren D, van Rijn RM, Burdorf A, Robroek SJW (2017). The influence of poor health on competing exit routes from paid employment among older workers in 11 European countries. Scand J Work Environ Health.

[CR23] Robroek SJW, Rongen A, Arts CH, Otten FWH, Burdorf A, Schuring M (2015). Educational inequalities in exit from paid employment among dutch workers: the influence of health, lifestyle and work. PloS one.

[CR24] Robroek SJW, Schuring M, Croezen S, Stattin M, Burdorf A (2013). Poor health, unhealthy behaviors, and unfavorable work characteristics influence pathways of exit from paid employment among older workers in Europe: a four year follow-up study. Scand J Work Environ Health.

[CR25] Rugulies R, Aust B, Madsen IEH, Burr H, Siegrist J, Bültmann U (2013). Adverse psychosocial working conditions and risk of severe depressive symptoms. Do effects differ by occupational grade?. Eur J Public Health.

[CR26] Schröder H, Kersting A, Gilberg R, Steinwede J (2013) Methodenbericht zur Haupterhebung lidA—leben in der Arbeit [Methodology Report of the main survey of lidA]. http://doku.iab.de/fdz/reporte/2013/MR_01-13.pdf. Accessed 15 Mar 2020

[CR27] Schuring M, Robroek SJW, Otten FWJ, Arts CH, Burdorf A (2013). The effect of ill health and socioeconomic status on labor force exit and re-employment: a prospective study with ten years follow-up in the Netherlands. Scand J Work Environ Health.

[CR28] Siegrist J (1996). Adverse health effects of high-effort/low-reward conditions. J Occup Health Psychol.

[CR29] Siegrist J, Starke D, Chandola T, Godin I, Marmot M, Niedhammer I, Peter R (2004). The measurement of effort–reward imbalance at work: European comparisons. Soc Sci Med.

[CR30] Stengård J, Leineweber C, Virtanen M, Westerlund H, Wang H-X (2022). Do good psychosocial working conditions prolong working lives? Findings from a prospective study in Sweden. Eur J Ageing.

[CR31] Tisch A (2015). Health, work ability and work motivation: determinants of labour market exit among German employees born in 1959 and 1965. J Labour Market Res.

[CR32] Toczek L, Bosma H, Peter R (2022). Early retirement intentions: the impact of employment biographies, work stress and health among a baby-boomer generation. Eur J Ageing.

[CR33] van den Berg T, Schuring M, Avendano M, Mackenbach J, Burdorf A (2010). The impact of ill health on exit from paid employment in Europe among older workers. Occup Environ Med.

[CR34] van den Berg TIJ, Elders LAM, Burdorf A (2010). Influence of health and work on early retirement. J Occup Environ Med.

[CR35] van Rijn RM, Robroek SJW, Brouwer S, Burdorf A (2014). Influence of poor health on exit from paid employment: a systematic review. Occup Environ Med.

[CR36] Virtanen M, Oksanen T, Batty GD, Ala-Mursula L, Salo P, Elovainio M, Pentti J, Lybäck K, Vahtera J, Kivimäki M (2014). Extending employment beyond the pensionable age: a cohort study of the influence of chronic diseases, health risk factors, and working conditions. PloS one.

