# Supplementary material for: Investigating the influence of work-related stress on early labour market exit: the role of health
Source: Eur J Ageing. 2023 Jul 5;20(1):31. doi: 10.1007/s10433-023-00778-7 (PMC10323059; doi:10.1007/s10433-023-00778-7)
Supplement: Supplementary file 1 — Additional file 1: Table S1 Distribution of socio-demographic and socio-economic indicators of the excluded individuals in % (n = 1089). [file 10433_2023_778_MOESM1_ESM.docx]

Additional file 1

**Table S1** Distribution of socio-demographic and socio-economic indicators of the excluded individuals in % (n = 1089).

| Variable | Distribution (in %) |
| --- | --- |
| Sex |  |
| Male | 42.0 |
| Female | 58.0 |
| Year of birth |  |
| 1959 | 44.8 |
| 1965 | 55.2 |
| Education |  |
| Low | 25.4 |
| Intermediate | 52.1 |
| High | 21.8 |
| Missing | 0.7 |
| Occupational status |  |
| Unskilled workers | 21.7 |
| Skilled workers | 34.1 |
| Middle management workers | 30.8 |
| Professionals | 4.2 |
| Missings | 9.3 |
| Income |  |
| Low | 27.3 |
| Middle-low | 34.8 |
| Middle-high | 15.3 |
| High | 7.7 |
| Missings | 14.9 |
